# Paediatric acquired demyelinating syndromes: incidence, clinical and magnetic resonance imaging features

**DOI:** 10.1177/1352458512445944

**Published:** 2013-01

**Authors:** Michael Absoud, Ming J Lim, Wui K Chong, Christian G De Goede, Katharine Foster, Roxana Gunny, Cheryl Hemingway, Philip E Jardine, Rachel Kneen, Marcus Likeman, Ken K Nischal, Michael G Pike, Naomi A Sibtain, William P Whitehouse, Carole Cummins, Evangeline Wassmer, The UK and Ireland Childhood CNS Inflammatory Demyelination Working Group

**Affiliations:** 1 School of Health and Population Sciences, University of Birmingham, Birmingham, UK.; 2 Department of Neurology, The Evelina Children’s Hospital at Guy’s and St Thomas’ NHS Trust, London, UK.; 3 Department of Neuroradiology, Great Ormond Street Hospital for Children, London, UK.; 4 Department of Paediatric Neurology, Royal Preston Hospital, Lancashire, UK.; 5 Department of Neuroradiology, Birmingham Children’s Hospital, Birmingham, UK.; 6 Department of Neurology, Great Ormond Street Hospital for Children, London, UK.; 7 Department of Neurology, Bristol Royal Hospital for Children, Bristol, UK.; 8 Department of Neurology, Alder Hey Children’s NHS Foundation Trust, Liverpool, UK; 9 Department of Neuroradiology, Bristol Royal Hospital for Children, Bristol, UK.; 10 Department of Ophthalmology, Great Ormond Street Hospital for Children, London, UK.; 11 Department of Neurology, Oxford Children’s Hospital, Oxford, UK.; 12 Department of Neuroradiology, King’s College Hospital NHS Trust, London, UK.; 13 School of Clinical Sciences, University of Nottingham, Nottingham, UK.; 14 Department of Neurology, Birmingham Children’s Hospital, Birmingham, UK.; * Joint last authors

**Keywords:** pediatric multiple sclerosis, paediatric multiple sclerosis, acute disseminated encephalomyelitis, demyelination, demyelinating, pediatric, paediatric, childhood, adolescent, epidemiology, incidence, magnetic resonance imaging

## Abstract

**Objective:**

Changing trends in multiple sclerosis (MS) epidemiology may first be apparent in the childhood population affected with first onset acquired demyelinating syndromes (ADSs). We aimed to determine the incidence, clinical, investigative and magnetic resonance imaging (MRI) features of childhood central nervous system ADSs in the British Isles for the first time.

**Methods:**

We conducted a population active surveillance study. All paediatricians, and ophthalmologists (*n* = 4095) were sent monthly reporting cards (September 2009–September 2010). International Paediatric MS Study Group 2007 definitions and McDonald 2010 MS imaging criteria were used for acute disseminated encephalomyelitis (ADEM), clinically isolated syndrome (CIS) and neuromyelitis optica (NMO). Clinicians completed a standard questionnaire and provided an MRI copy for review.

**Results:**

Card return rates were 90%, with information available for 200/222 positive notifications (90%). After exclusion of cases, 125 remained (age range 1.3–15.9), with CIS in 66.4%, ADEM in 32.0% and NMO in 1.6%. The female-to-male ratio in children older than 10 years (*n* = 63) was 1.52:1 (*p* = 0.045). The incidence of first onset ADS in children aged 1–15 years old was 9.83 per million children per year (95% confidence interval [CI] 8.18–11.71). A trend towards higher incidence rates of ADS in children of South Asian and Black ethnicity was observed compared with White children. Importantly, a number of MRI characteristics distinguished ADEM from CIS cases. Of CIS cases with contrast imaging, 26% fulfilled McDonald 2010 MS diagnostic criteria.

**Conclusions:**

We report the highest surveillance incidence rates of childhood ADS. Paediatric MS diagnosis at first ADS presentation has implications for clinical practice and clinical trial design.

## Introduction

Multiple sclerosis (MS) is a chronic disabling and costly neurodegenerative disorder. Incidence of MS appears to be increasing worldwide with a rising female-to-male sex ratio,^1,2^ and with approximately 5% of adults who develop MS having first symptoms in childhood.^3,4^ Some epidemiological evidence suggests early environmental exposures may influence MS risk.^5,6^ Hence, any epidemiological change in MS may be evident first in a paediatric population.

Diagnosis of MS in children, as in adults, is first heralded by onset of a clinically isolated syndrome (CIS), or more rarely acute disseminated encephalomyelitis (ADEM). Children diagnosed with relapsing–remitting MS present with one of the central nervous system (CNS) acquired demyelinating syndromes (ADS) followed by a second event usually within 2 years.^7^ Childhood CIS presentations with an abnormal magnetic resonance imaging (MRI) of the brain (i.e. more than one high T2 signal) compared with ADEM are more likely to have relapsing disease.^8-12^ Recently, the new MRI McDonald 2010 MS criteria have been recommended for use in children presenting with a CIS,^13^ potentially allowing for MS diagnosis at first presentation of a CIS.^14^ There is no biomarker that differentiates between monophasic ADEM, monophasic CIS, or MS, hence a combination of clinical, cerebrospinal fluid (CSF) positive oligoclonal bands and MRI features is suggested.^12, 15^ MRI differences, in particular between ADEM and polyfocal CIS, are also not well recognized.^16^ The differential diagnosis of ADS is complex and wide, especially so in childhood.^17^

The International Paediatric Multiple Sclerosis Study Group (IPMSSG) have published consensus definitions (2007) of paediatric CNS ADS and MS to facilitate uniformity in clinical practice and future epidemiological research.^4^ Two previous surveillance studies in Canada (2004–2007) and Germany (1997–1999) revealed an incidence ranging from 3.7^18^ to 9.0 per million children per year.^19^ The incidence of childhood ADS remains largely unknown worldwide, and as the incidence of these disorders based on the epidemiology of MS in adults is likely to differ geographically, estimates are needed from multiple geographical regions.

We report a multinational multisource^20^ prospective active surveillance study in the British Isles (latitudes 50–59°N) to ascertain incidence, clinical, investigative and MRI features of childhood (age <16 years) ADS using panel review to apply IPMSSG consensus definitions (including the incorporated McDonald MRI 2001 and Barkhof space diagnostic criteria).^4, 21^ We also apply the newly revised 2010 McDonald space and time diagnostic criteria at first ADS presentation;^13^ and the recent Verhey MRI prognostic MS criteria.^22^

## Methods

### Study design

We carried out a prospective active surveillance study of first episode ADS in children aged between 1 and 15 years 11 months of age, in the British Isles. We used multisource ascertainment via two well-established surveillance units in order to establish incidence of new cases.

### The British Paediatric Surveillance Unit (BPSU), the primary source of data

The British Paediatric Surveillance Unit (BPSU) facilitates epidemiological surveillance of childhood diseases of public health importance in the UK and Republic of Ireland. The BPSU, launched in 1986, has helped initiate over 80 studies with a card return rate consistently higher than 93%.

### The British Ophthalmological Surveillance Unit (BOSU), the alternative source of data for optic neuritis cases

The British Ophthalmological Surveillance Unit (BOSU) enables ophthalmologists to participate in the nationwide surveillance of uncommon ophthalmological conditions. The BOSU card return rate is consistently higher than 75%.

### Case ascertainment via active surveillance

One month before commencing the study, a protocol card and letter was sent to all clinicians informing them of the study. Monthly notification cards (mean 4095/month) were sent by the BPSU (mean 2945/month) to all registered certified consultant paediatricians and paediatric neurologists and BOSU (mean 1150/month) to all registered ophthalmologists (between September 2009 and September 2010). In the British Isles, these clinicians are likely to see all cases of childhood ADS. Clinicians returned the card to the surveillance units notifying any cases or confirming ‘nothing to report’. Upon receipt of a positive notification the surveillance units provided the investigating team with a BPSU/BOSU case number and clinician contact details. The study team contacted the clinician directly, sending a detailed data collection form and a blank CD for an anonymized MRI. As more than one clinician might notify the same patient seen in different settings, minimal identifiers (National Health Service [NHS] number, district postcode, sex and date of birth) were used for record linkage and to exclude duplicates with a high level of certainty (http://www.rcpch.ac.uk/bpsu).

### Initial surveillance reports

Using the IPMSSG consensus definitions for classification of paediatric ADS,^4^ we asked clinicians to report children experiencing clinical neurological events consistent with a first episode ADS and confirmed with white matter changes (except in optic neuritis) on MRI (Table 1).

**Table 1.** Summarized inclusion definitions for CNS acquired demyelinating syndromes.

Condition	Definition
Acute disseminated encephalomyelitis (ADEM)	(1) A polysymptomatic clinical event with acute/subacute onset that must include encephalopathy (behavioural change or altered consciousness). (2) MRI brain shows multifocal lesions.
Clinically isolated syndrome (CIS)	A first acute-clinical episode of CNS symptoms which may either be monofocal or multifocal, but does not include encephalopathy (except in brainstem syndromes). The MRI will show white matter demyelination. These include: 1. Transverse myelitis (TM): weakness and/or numbness of both legs +/- arms, usually with maximal deficits 1 week after symptom onset supported by demyelination on MRI spine. 2. Optic neuritis: Acute or subacute loss of vision and ≥1 of: relative afferent pupillary defect (unilateral cases), visual field deficit or scotoma, impaired colour vision, optic disc oedema, or abnormal visual evoked potentials. MRI is not necessary for diagnosis. 3. Other CIS: Brainstem, cerebellar, and/or hemispheric dysfunction, supported by demyelination on MRI.
Neuromyelitis optica (NMO)	Must have: i. Optic neuritis and ii. Acute myelitis. Must have: iii. Spinal MRI lesion extends over three or more segments or iv. Aquaporin-4 antibody testing is positive. The brain MRI may be abnormal but must not meet Multiple Sclerosis MRI diagnosis criteria.
Exclusion criteria	1. Leukodystrophies (e.g. metachromatic leukodystrophy, adrenoleukodystrophy) or mitochondrial disease. 2. Proven CNS infection (e.g. viral encephalitis, bacterial meningitis, herpes simplex encephalitis, Lyme disease, HIV). 3. Radiation/chemotherapy associated white matter damage. 4. Condition fulfilling criteria for CNS connective tissue disease e.g. lupus, vasculitis. The sole presence of antibodies associated with CNS connective tissue or autoimmune diseases was not sufficient for exclusion.

### Data collection

A condition specific targeted neurological history was collected at the time of first demyelinating event. Data collected included: basic demographic data; demyelinating symptoms and signs; CSF findings, serological and microbiological results; timing of MRI being carried out and whether abnormal; and treatment given.

### MRI imaging review

MRI scans were reviewed blinded to clinical features by MA and one of five neuroradiologists (KC, KF, RG, ML, NS). A standardized proforma was completed utilizing previously described nomenclature (supplementary material appendix 1).^10, 23, 24^ McDonald 2001 dissemination in space criteria (as recommended by the current IPMSSG criteria),^4, 21^ McDonald 2010 dissemination in space and time (if a gadolinium-enhanced scan was available) criteria and Verhey prognostic criteria were classified by MA according to the MRI variables and associated panel review.^13^ Features of ADEM and CIS scans were compared. Although a single MRI protocol was not feasible as this was a national population based study collecting data from established clinical practice from all centres, all MRI brain scans were performed on 1.5 T scanners and had a minimum of a T1-weighted and a T2-weighted or fluid-attenuated inversion recovery (FLAIR) sequence in two different planes. Additional sequences included gadolinium-enhanced T1, diffusion weighted and spine images. MRI slice thickness varied from 3–5 mm.

### Panel classification and agreement for inclusion

The co-investigators met quarterly as a panel to review reported cases (at least four members present at each meeting). The panel consisted of paediatric neurologists (*n* = 8; CDG, CH, EW, MJL, MP, PJ, RK, WPW) with expertise in ADS, and final classification as per IPMSSG clinical criteria was arrived at by consensus. MRI images (with neuroradiologist scoring) were used to confirm the presence of lesions consistent with inflammatory demyelination, to aid in the exclusion of cases and to sub-classify CIS cases with asymptomatic MRI lesions (supplementary material appendix 1).^17^

### Statistical analysis

Descriptive statistics were used to summarize the key components of the dataset. Estimates of national incidence with confidence intervals (Byar’s approximation of the exact Poisson) for the 13-month study was annualized using mid-2010 UK and 2010 Republic of Ireland population estimates.^25,26^ Mid-2009 population estimates by ethnicity for England and Wales were used to evaluate rates of childhood ADS by ethnicity (Table 2). A sensitivity analysis was conducted on a worst-case scenario that all reported cases with missing clinical histories were actually cases of ADS to assess the potential impact on estimated incidence of ADS. We used a two source case capture–recapture analysis to estimate missed cases of optic neuritis on the assumption of independence,^27^ but did not carry out capture–recapture analysis for other ADS, as referral networks mean that the assumption of independence was violated. The England 2010 index of multiple deprivation was used to derive the percentage of cases in England living in the 20% most deprived districts.^28^ Parametric or non-parametric statistical tests (Kruskal–Wallis tests) were used for continuous distributions as appropriate given normality and χ^2^ or Fisher’s exact tests for nominal data. Agreement between reporters and the panel was measured using the Kappa statistic. Statistical analysis was performed using PASW Statistics for Windows version 17.0 (© SPSS, Inc., 2009, Chicago, IL, www.spss.com) and the openepi software (version 2.3.1, www.openepi.com).^29^

**Table 2.** Incidence of childhood CNS acquired demyelinating syndromes by country, sensitivity analysis and ethnic group in England and Wales.

CONFIRMED CASES						
Region	Number	Percentage	Population 2010 1–15 years (per thousands) or %	Incidence per annum per million	95% CI lower	95% CI upper
England, Wales and Channel Islands	117	93.6	9636	11.2	9.27	13.4
Scotland	3	2.4	853	3.25	0.65	9.49
Ireland and Northern Ireland	5	4.0	1254	3.68	1.19	8.59
Total	125	100.0	11743	9.83	8.18	11.71
**Sensitivity Analysis Assuming All Reports With Missing Information Are Cases**						
England, Wales and Channel Islands	129	87.8	9636	12.36	10.32	12.68
Scotland	9	6.1	853	9.74	4.44	18.5
Ireland and Northern Ireland	9	6.1	1254	6.63	3.0	12.6
Total	147	100.0	11743	11.6	9.76	13.6
**Incidence By Ethnicity In England And Wales**						
White	93	80.17%	83.93%	10.62	8.57	13.00
South Asian: Indian; Pakistani; Bangladesh; other South Asian background	11	9.48%	7.30%	14.43	7.20	25.83
Black	7	6.03%	3.31%	20.25	8.12	41.73
Chinese, mixed and other	5	4.31%	5.46%	8.78	2.82	20.27
Total	116	116	100%			

### Approvals

The study was approved by the BPSU and BOSU executive committees. Data handling was restricted to those with direct involvement in the project. To comply with collection of minimal identifier information regulations without patient consent as in Section 251 of the NHS Act 2006, the study has approval from the National Information Governance Board’s Ethics & Confidentiality Committee (ECC/BPSU 4-03[FT1]/2009) and the UK Multicentre Research Ethics Committee (09/H1202/92).

## Results

### Card return rate, expert review and record linkage outcome

**Figure 1.**
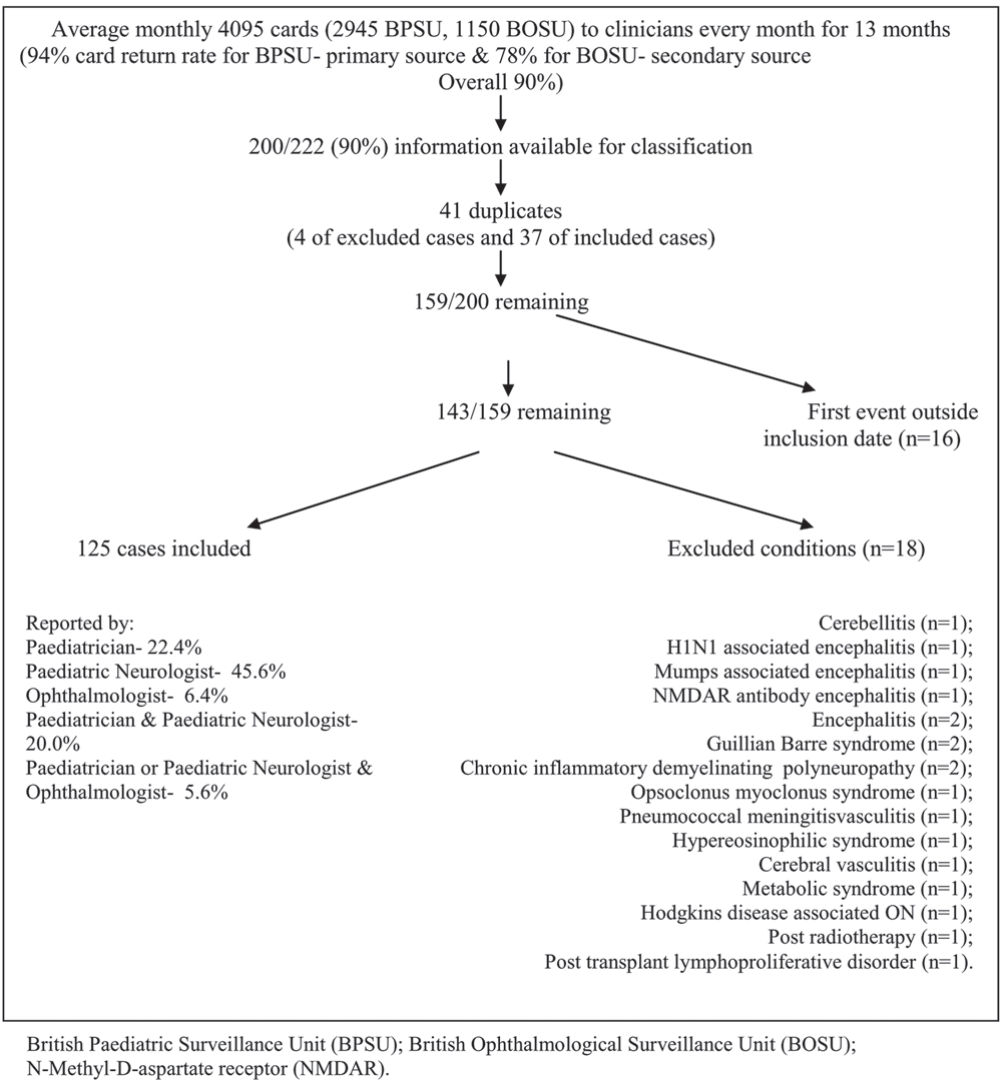
Study flow chart showing panel review and record linkage outcome.

Card return rate was 94% from the BPSU and 78% from the BOSU (total 47,910 from 53,235 cards returned including negative reports). There were 222 positive notifications (197 BPSU and 25 BOSU) with insufficient information for review of 22 notifications (6 Scotland, 12 England and Wales, and 4 Ireland and Northern Ireland). In the remaining 200 (90%), 41 duplicate notifications were identified. Cases where the event was outside the inclusion dates, September 2009 to September 2010 (*n* = 16) were excluded. A further 18 cases were excluded by the panel (Figure 1). A total of 124/125 cases remaining had MRI scans (one isolated optic neuritis had no MRI scan, four transverse myelitis cases had MRI spine only).

### Incidence of childhood CNS inflammatory demyelination in the British Isles

The minimum incidence of first onset ADS in children aged 1–15 years old in the British Isles is 9.83 per million children per year (95% confidence interval [CI] 8.18–11.71). Incidence was highest in England and Wales (Table 2). A sensitivity analysis showed no significant difference in incidence rates within countries in the British Isles.

### Demographic characteristics

Median age was 10.0 years (range 1.3–15.9; mean 9.74 years, SD 4.34) and 51.2% (*n* = 64) of cases were female (Table 3). The median age for males was 8.9 years (interquartile range [IQR] 5.0–13.5) and for females was 11.4 years (IQR 6.8–14.4; *p* = 0.046). The female-to-male ratio in children younger than 10 years (*n* = 62) was 0.72:1 and in older than 10 years (*n* = 63) was 1.52:1. White children comprised 81% of the cohort. Higher incidence rates of ADS in children of South Asian and Black ethnicity in England and Wales compared with White children were observed, although not reaching statistical significance (Table 2). A total of 34% of the cases in England lived in the most 20% deprived districts. Children living in the most 20% deprived areas were more likely to be males compared with female (23:13) when compared with those living in the 80% least deprived areas (30:40, *p* = 0.040). They were also most likely to be of non-White ethnicity (12:24 versus 11:58, *p* = 0.041). Age and ADS presentation did not influence those living in the most 20% deprived areas.

**Table 3.** Clinical, demographic, and phenotypic expert panel classification of the childhood CNS acquired demyelinating syndromes.

		CIS (*n* = 83)				
		ADEM (*n* = 40)	ON (*n* = 31)	TM (*n* = 26)	Other CIS (*n* = 26)	NMO (*n* = 2)
**Age at presentation: median (IQR)**		5.3 (3.6-7.0)	11.8 (9.0-13.9)	12.6 (9.3-14.0)	14.0 (9.5-14.5)	6.4 and 14.8 years
**Sex (*n* = 125)**	**Male: Female (% female) 61: 64 (51%)**	24:16 (40%)	15:16 (52%)	11:15 (58%)	11:15 (58%)	0:2 (100%)
		37:46 (55%)				
**Ethnicity (*n* = 124/125)**	**White (*n* = 101)**	36 (90%)	21 (68%)	22 (85%)	21 (81%)	1 (50%)
	**Asian (*n* = 11)**	3	4	2	1	1
	**Black (*n* = 7)**	1	3	1	2	0
	**Chinese, mixed, other (*n* = 5)**	0	3	1	1	0
**Clinical:**	**MRI:**					
Monofocal	≥ 1 asymptomatic lesion		7 (23%)	9 (35%)	4 (15%)	
	no asymptomatic		24	17	3 (12%)	
Multifocal	≥ 1 asymptomatic		0	0	16 (62%)	
	no asymptomatic		0	0	2 (8%)	
Radiologically isolated			n/a	n/a	1	
**Spinal lesion(s) present: *n*** (% ≥ 3 segments)		8/12 (100%)	1/8 (0)	26/26 (62%)	4/9 (75%)	2/2 (100%)
**Death**		1/40 (2.5%)	0	0	0	0
**Seizures**		8/40 (20%)	0	0	0	0
**Optic neuritis:** Unilateral: bilateral (% bilateral)		0:3 (100%)	22:9 (29%)	0:0	0:1	1:1 (50%)
**Cerebellar signs: *n* (%)**		18 (45%)	0	0	11 (42%)	0
**Brain stem signs: *n* (%)**		11 (28%)	0	1	13 (50%)	0
**Pyramidal signs: *n* (%)**		24 (60%)	0	0	10 (38%)	0
**Intensive care unit admissions**; *n* = 12 (10%)		8 (20%)	0/31 (0%)	3/26 (12%)	1/26 (4%)	0/2
**Plasma exchange**; *n* = 4 (3%)		2 (5%)	0/31	2/26 (8%)	0/26	0/2
**Intravenous corticosteroids**		37/40 (93%)	23/31 (74%)	25/26 (96%)	18/26 (69%)	2/2 (100%)
**Oral steroids**		27/40 (68%)	21/31 (67%)	16/26 (62%)	11/26 (42%)	2/2 (100%)
**Intravenous immunoglobulin**		6/40 (15%)	0	7/26 (27%)	1/26 (4%)	0

ADEM, acute disseminated encephalomyelitis; ON, optic neuritis; TM, transverse myelitis; CIS, clinically isolated syndrome; NMO, neuromyelitis optica; MRI, magnetic resonance imaging; IQR, interquartile range.

### Results of panel review and clinical classification

**Figure 2.**
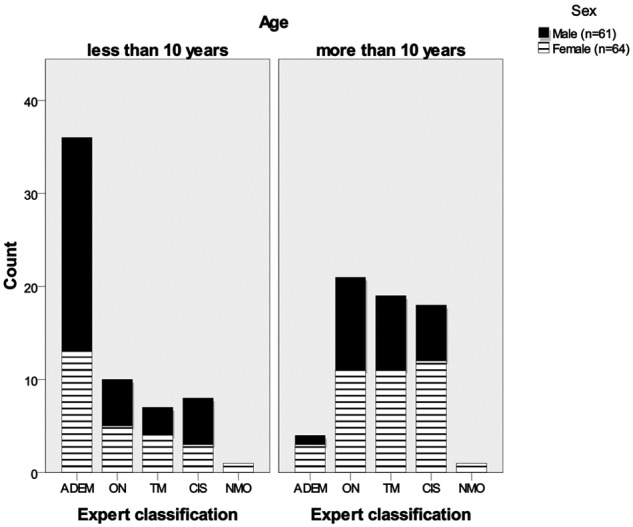
Childhood acquired demyelinating syndromes by age (younger and greater than 10 years old) and sex.

Childhood ADS were classified as CIS in 66.4%, ADEM in 32.0% and neuromyelitis optic (NMO) in 1.6%. Of the CIS cases, optic neuritis (ON) represented 37.4%, transverse myelitis (TM) 31.3% and other CIS 31.3% (Table 3). The majority (70%) of the other CIS cases were polysymptomatic. Children with ADEM were younger than cases presenting with CIS (*p* < 0.001) (Figure 2). The risk ratio for CIS in females compared with males was 1.46 (95% CI 1.17–1.83). The panel reclassified a total of 15 out of the 125 cases (12%): ADEM to CIS (*n* = 11) mainly due to lack of encephalopathy; Other CIS to TM (*n* = 3); TM to other CIS (*n* = 1). The overall level of agreement between reporters and the panel was good (Kappa 0.838 [95% CI 0.761–0.914]).

### Deaths

Clinicians reported three deaths in the initial hospital admission, one in a child with ADEM and two in cases which proved not to have ADS (post-transplant lymphoproliferative disorder and hypereosinophilic syndrome).

### Capture–recapture analysis

Capture–recapture analysis for ON cases (*n* = 31) assuming that BPSU and BOSU were independent sources of cases estimated the total population to be 47 (95% CI 30–55).

### MRI features

More CIS cases fulfilled MRI McDonald 2010 dissemination in space criteria (42%) compared with the 2001 criteria (28%; Table 4). Of the CIS cases which had contrast-enhanced scans (*n* = 38), 10 cases (26%) fulfilled McDonald 2010 space and time criteria for MS diagnosis. Verhey MRI prognostic criteria for identifying childhood ADS at high risk of MS (presence of one T1 hypointense and one periventricular lesion) were fulfilled in 11/78 (14%) CIS cases compared with 1/40 (2.5%) ADEM cases. A total of 19/78 (24%) CIS cases fulfilled either Verhey prognostic criteria or McDonald 2010 MS space and time criteria for MS (8/10 cases which fulfilling McDonald 2010 space and time criteria did not fulfil Verhey criteria, whilst 3/5 fulfilling Verhey criteria did not fulfil McDonald criteria).

**Table 4.** MRI criteria fulfilled.

	Panel classification		
	CIS (*n* = 83)		
	ON (*n* = 31)	TM (*n* = 26)	Other CIS (*n* = 26)
**McDonald 2001 space criteria**	4/30	5/22	13/26
	22/78 (28%)		
**McDonald 2010 space criteria**	5/30	8/22	20/26
	33/78 (42%)		
**McDonald 2010 time criteria (gadolinium given scans)**	0/12	3/11	7/15
	10/38 (26%)		
**Meets McDonald MRI criteria for MS on initial presentation (dissemination in space and time)**	10/38 (26%)		
**Verhey or McDonald 2010 MS criteria present**	19/78 (24%)		

MRI, magnetic resonance imaging; MS, multiple sclerosis; ON, optic neuritis; TM, transverse myelitis; CIS, clinically isolated syndrome.

ADEM cases compared with CIS cases with abnormal MRI (supplementary material appendix 2) were more likely to have more deep grey nuclei (*p* < 0.001), large white matter (*p* < 0.001) and cortical grey matter (*p* = 0.001) high T2 signal lesions. CIS scans were more likely to have more periventricular (*p* < 0.001), deep white matter (*p* = 0.003), corpus callosum (*p* = 0.001), well-defined and discrete high T2 signal lesions (*p* < 0.001) and black holes (*p* = 0.008). The number of total high T2 signal, enhancing, infratentorial, juxtacortical and spinal lesions did not differentiate between CIS and ADEM patients. Of the ON and TM cases (monofocal CIS) 23% and 35% had one or more high T2 signal brain lesion respectively (Table 3).

### Investigative features

Children with ADEM more commonly had CSF lymphocytosis (*p* = 0.005) and less commonly CSF positive oligoclonal bands (*p* = 0.011) compared with those with CIS (Table 5). One ADEM case was reported as having the common missense mutation (c.1880CT, p.Thr585Met) of the Ran Binding Protein 2 (RANBP2) gene previously reported in recurrent or familial acute necrotising encephalopathy.30 Of those tested for the aquaporin-4 antibody (n = 52), none of the ADEM (n = 5), TM (n = 20), and other CIS (n = 7) cases tested positive while one child with ON and both NMO patients tested positive.

**Table 5.** Investigative features.

	Expert panel classification				
	ADEM (*n* = 40)	CIS (*n* = 83)			NMO (*n* = 2)
		ON (*n* = 31)	TM (*n* = 26)	Other CIS (*n* = 26)	
**CSF oligoclonal bands**	2/20 (10%)	6/17 (35%)	6/19 (32%)	10/15 (67%)	0/1 (0%)
**CSF WBC ≥5 × 10^6^/l**	21/36 (58%)	3/17 (18%)	8/20 (40%)	8/21 (38%)	0/2 (0%)
**CSF protein median (IQR)**	0.38 (0.25–0.58)	0.26 (0.23–0.35)	0.36 (0.26–0.52)	0.29 (0.21–0.36)	0.29
	*n* = 28	*n* = 17	*n* = 19	*n* = 17	*n* = 2
**Genetic tests reported**	1 case RANBP2 positive	n/a	n/a	n/a	n/a

ADEM, acute disseminated encephalomyelitis; ON, optic neuritis; TM, transverse myelitis; CIS, clinically isolated syndrome; NMO, neuromyelitis optica; CSF, cerebrospinal fluid RANBP2, Ran Binding Protein 2 gene; IQR, interquartile range; WBC, white blood cell.

### Treatment

The majority (89%) received either intravenous corticosteroids (84%) and/or oral prednisolone in the first instance. A higher proportion of children with ADEM were required intensive care unit (ICU) admission, and four children (two ADEM and two TM) received plasma exchange. Intravenous immunoglobulin was administered to 11% of the cohort (Table 3).

## Discussion

The minimum incidence of first onset childhood ADS in the British Isles is 9.83 per million (95% CI 8.18–11.71) children per year aged 1–15 years. This is the highest reported incidence of childhood ADS from a prospective childhood surveillance study. To date, there have been two surveillance studies studying ADS. A Canadian surveillance study (2004–2007) estimated the incidence of childhood ADS to be 9.0 per million people aged less than 18 years old,^19^ with 22% classified as ADEM and 78% as CIS. Although this study did not have MRI review and was conducted prior to the IPMSSG consensus definitions, it is unlikely that these factors would have had a major influence on classification. An earlier surveillance study from Germany (1997–1999) estimated the incidence of MS in children <16 years as 3.0 per million children, and that of ADEM as only 0.7 per year per million children aged less than 16 years old.^18^ This study asked clinicians to report new cases of definite or suspected MS or ADEM cases, and did not include other ADS. Diagnosis of MS and ADS depends on clinical definitions and MRI criteria used, and hence it may be that these ascertainment estimates would change with current definitions and criteria (supplementary material appendix 3).

The mean age of onset (9.7 years) was similar to the Canadian incidence study (mean 10.5 years) and a French^9^ (children <16 years, 1985-2001) first episode ADS retrospective cohort study (mean 9.9 years), but older than a Dutch^10^ (children <16 years, 1990–2010) retrospective cohort study (8.5 years). A recent retrospective study from the USA (*n* = 85) showed the incidence of childhood ADS and MS to be higher in Black children compared with White and Hispanic children.^31^ While non-White ethnic groups were over-represented in our study (*n* = 125), this did not reach statistical significance. Although there is limited power to compare subgroups in the study, this still contrasts with the adult MS population where white ethnic groups are over-represented. Adult and paediatric^7,14,32^ MS studies have shown that adolescent females are over-represented compared with males with ratios of up to 3:1. An adult CIS study has also shown a higher risk ratio of females developing CIS,^33^ the first presentation of MS. Our study shows that in childhood first onset ADS, sex ratios also varied with age, with more females in the older age groups (F:M ratio of 1.52:1 in children over 10 years). Children older than 10 years more often had CIS compared with those less than 10 years of age who presented more often with ADEM. A Japanese retrospective multicenter study showed the male-to-female ratio of ADEM to be 2.3:1, and that ADEM was three times more common than MS.^34^ In addition previous UK epidemiological adult studies have shown that MS hospital admissions, incidence and prevalence were higher in those living in least deprived areas. ^35,36^ Our study showed that children with ADS living in the most 20% deprived areas (in England) were more likely to be males and of non-White ethnicity. These observations regarding the geographical deprivation indices and demographics of childhood ADS (over-representation of older females with CIS, and children with Black and South Asian ethnicity) add further support to the hypothesis that environmental influences may indeed be acting as early as in childhood.

This study is not without limitations. As with other epidemiological studies, some cases may not have been reported or have had sub-clinical presentations, and it is plausible that children in our study who met McDonald 2010 criteria for MS at first clinical presentation may have had previous symptoms not identified as ADS. This was addressed as far as was possible by using clear consensus case definitions and multiple sources of case ascertainment. A capture–recapture analysis estimated that a possible 16 ON cases were missed out of a potential 47, although this relies on assumptions of complete independence that may not hold in practice.

Our study shows that childhood CIS occurs more often than ADEM (ratio 2.1:1) and also confirms a previous report that ADEM can be a severe disease with one death and one in five requiring intensive care admission.^37^ The proportion of children with ADEM in our cohort (32%) was slightly higher than French^9^ (29%), Canadian (22%), and Dutch^10^ cohorts (termed polyfocal ADS with encephalopathy, not ADEM).

We found that 43% of CIS patients had one or more asymptomatic brain lesion. Previous studies have shown that children with CIS with abnormal brain scans are at a higher risk of relapse than those without asymptomatic brain lesions.^11,12^ Also it is of interest that a number of MRI measures significantly helped differentiate ADEM from CIS cases. For example, ADEM cases compared with CIS had more deep grey nuclei and cortical grey matter high T2 signal lesions. CIS cases had more periventricular, deep white matter, corpus callosum high T2 signal lesions and black holes.

CSF lymphocytosis and negative oligoclonal bands were associated with an ADEM diagnosis, in contrast to a previous study.^16^ It is of interest that one case was reported as positive for the common mutation in the RANBP2 gene, which has been recently described in familial or recurrent acute necrotizing encephalopathy (ANE).^30^ With the increasing recognition of the overlap between the MRI and clinical features of ADEM and ANE, and further reports of other genetically predetermined encephalopathies,^38^ this potential novel group of genetic determinant(s) of encephalopathy in demyelination requires exploration.

A total of 26% of CIS cases who had gadolinium given scans (*n* = 38) fulfilled McDonald 2010 MRI criteria for MS at onset (space and time), hence a minimum 12% of CIS patients met McDonald 2010 MS diagnostic criteria at diagnosis. In addition 24% of CIS cases fulfilled either McDonald 2010 or Verhey criteria which are thought to indicate higher risk of MS, although both the McDonald criteria and the Verhey model require further validation in prospective multinational cohorts. This is particularly the case as in this study 8/10 of cases fulfilling McDonald 2010 MS criteria did not meet the criteria from Verhey’s predictive model. We have previously shown that 61% of children eventually diagnosed with MS and who had contrast-enhanced scans at ADS onset fulfilled McDonald 2010 MS criteria at first scan.^14^ Not all children in this study had spinal imaging and contrast administered, and MRI sequence variation could have affected the sensitivity of MRI.

The potential to diagnose MS at disease onset has implications for MRI protocols for children with suspected ADS, and individual patient treatment decisions. This may be particularly important as new MS drugs are emerging with reported better efficacy in adults than current injectables, and the simultaneous requirement for new drugs to undergo testing in children with the aim of ensuring robust evidence to support authorization by regulatory agencies. To date, eight new MS drugs have been recommended for a Paediatric Investigation Plan,^39^ hence earlier diagnosis may increase the eligible clinical trial population. Prognostic and outcome data are needed to inform these decisions. A prospective 2-year follow up of this patient cohort is under way and a further multicentre cohort study with detailed outcome measurements and a biobank have been established.^40^ These should yield prognostic information and aid validation of consensus definitions, and the clinical utility of the new McDonald and Verhey proposed MRI criteria for early diagnosis of MS in children.
